# Correlation analysis of omega-3 fatty acids and mortality of sepsis and sepsis-induced ARDS in adults: data from previous randomized controlled trials

**DOI:** 10.1186/s12937-018-0356-8

**Published:** 2018-05-31

**Authors:** HuaiSheng Chen, Su Wang, Ying Zhao, YuTian Luo, HuaSheng Tong, Lei Su

**Affiliations:** 1 Department of Critical Care Medicine, Guangzhou School of Clinical Medicine, Southern Medical University (Guangzhou General Hospital of Guangzhou Military Region), No.111 Liuhua Road, Guangzhou, 510010 China; 2 Department of Critical Care Medicine, Shenzhen People’s Hospital/Second Clinical Medical College of Jinan University, Shenzhen, Guangdong China; 3 Department of Cardiovascular and thoracic Surgery, PLA 305 Hospital, A-13 Wenjin Street, Xicheng, Beijing, 100017 China; 4 Department of Critical Care Medicine, Institute: Shenzhen People’s Hospital/The Second Clinical Hospital of Jinan University, No.1017, Dong Men North Road, Luohu District, Shenzhen City, 518020 China

**Keywords:** Sepsis, Multiple organs dysfunction syndrome, Acute respiratory distress syndrome, Omega 3 fatty acid, Mortality

## Abstract

**Objective:**

This study aimed to investigate the possible effect of omega-3 fatty acids on reducing the mortality of sepsis and sepsis-induced acute respiratory distress syndrome (ARDS) in adults.

**Methods:**

Medline, Embase, Cochrane Library, China National Knowledge Infrastructure (CNKI) database, WangFang database, and Chinese BioMedical Literature Database from their inception to March 6, 2017, were searched using systematic review researching methods. Five factors were analyzed to investigate the correlation between omega-3 fatty acids (either parenteral or enteral supplementation) and mortality rate.

**Results:**

Forty randomized controlled trials (RCTs) were initially included, but only 25 of them assessed mortality. Of these RCTs, nine used enteral nutrition (EN) and 16 used parenteral nutrition (PN). The total mortality rate in the omega-3 fatty acid group was lower than that in the control group. However, the odds ratio (OR) value was not significantly different in the EN or PN subgroup. Eighteen RCTs including 1790 patients with similar severity of sepsis and ARDS were also analyzed. The OR value was not significantly different in the EN or PN subgroup. Omega-3 fatty acids did not show positive effect on improving mortality of sepsis-induced ARDS (*p* = 0.39). But in EN subgroup, omega-3 fatty acids treatment seemed to have some benefits in reducing mortality rate (*p* = 0.04). In the RCTs including similar baseline patients, partial correlation analysis found that the concentration ratio of *n*-6 to *n-3 fatty acids* had positive correlation with reduction of mortality (RM) (*γ* = 0.60, *P* = 0.02), whereas the total number of each RCT had negative correlation with RM (*γ* = − 0.54, *P* = 0.05).

**Conclusions:**

This review found that omega-3 fatty acid supplementation could reduce the mortality rate of sepsis and sepsis-induced ARDS. However, further investigation based on suitable concentrations and indications is needed to support the findings.

## Background

Sepsis is a critical infectious condition that can cause immune system response and organ dysfunction [1]. Antibiotics, fluid resuscitation, and organ support measurements can be used to improve clinical outcomes according to the guideline on commencement of sepsis [2, 3]. Despite worldwide efforts to improve the outcomes, the mortality rates of severe sepsis and sepsis shock were 25%–30% and 40%–70%, respectively [4, 5].

The immune response is supposed to be correlated with sepsis [6]. Two phases have been described in sepsis: an initial systemic inflammatory response syndrome followed by the negative feedback of a secondary compensatory anti-inflammatory response syndrome [7]. In the early stage of the disease, immunosuppression was found although the systemic inflammatory reaction was dramatic. The immune dysfunction of CD4+ T cells is the primary cellular mechanism behind sepsis and sepsis-induced multiple organs dysfunction syndrome (MODS) [8]. The ratio of CD4 to CD8 has long been supposed to represent T lymphocyte function, which was negated by recent consensus [9]. Several studies discussed that omega-3 fish oil or omega-3 fatty acids could improve the ratio of CD4 to CD8 in patients with severe sepsis to reduce the mortality [10]. However, recent studies suggested that regulatory T lymphocytes (Tregs), but not the ratio of CD4+/CD8+, could represent the immune function status of sepsis [11]. Randomized controlled trials (RCTS) also found that omega-3 fatty acids could improve CD4 + CD25+ Tregs in the blood of patients with sepsis, which was a marker of improving patient’s immune function [12]. In fact, omega-3 fatty acids could also enhance the immune effects via other avenues, such as inhibiting the production of inflammatory factors [13], preventing the nuclear factor (NF)-kappaB pathway, inhibiting the activity of Toll-like receptor 4 (TLR4), and blocking the TLR signal transduction [14].

Fatty acids can be divided into essential and non-essential fatty acids [15]. The former mainly includes omega-6 and omega-3 fatty acids. All kinds of fatty acids are involved in regulating inflammatory responses with different effects [16]. When inflammation occurs, arachidonic acid (AA) on the cell membrane produces lipid mediators with a series of physiological activities, including prostaglandin E2 (PGE2) and leukotriene 4 (LT4), both of which promote the accumulation of leukocytes and accelerate the release of pro-inflammatory cytokines tumor necrosis factor alpha and interleukin 1 [17]. Eicosapentaenoic acid (EPA) and docosahexaenoic acid (DHA) were two main components of polyunsaturated fatty acids [18]. EPA could compete with the same enzyme system of AA to produce prostaglandin E3 (PGE3) and leukotriene 5 (LT5) rather than PGE2 and LT4 [19]. Both products of EPA had no effects on inflammatory activity. Polyunsaturated fatty acid is also an important component of cell membrane phospholipids, which affects the stability of the cell membrane [20]. It changes membrane lipid rafts, replaces the *n*-6 polyunsaturated fatty acids (PUFA) of the inflammatory cell membrane with *n*-3 PUFA, reduces the production of arachidic acid from *n*-6 PUFA, protects the fluidity of CD4+ lymphocyte membrane, and stabilizes the cell membrane [19]. All these researches supported the viewpoint that omega-3 fatty acids had anti-inflammatory effects and improved cellular immune function in patients with sepsis to reduce the mortality [14]. However, this conclusion is still controversial.

A randomized controlled trial including 30 patients with sepsis was conducted in 2011 [21]. Patients were assigned to omega-3 fish oil fatty acid application group and control group. Other therapeutics was similar in both groups. The mortalities were not significantly different. In this study, some patients with advanced malignant tumours were included, which might affect the final outcomes. After that, two other RCTs were designed, both of which included patients with severe sepsis-induced gastrointestinal dysfunction [22, 23]. One patient with gastrointestinal dysfunction was assessed according to China criteria [22], while the other patients were assessed using the criteria made by the Europe Working Group on Abdominal Problems (WGAP) [23, 24]. Although both RCTs included patients with severe sepsis, the mortality between trials was insignificantly different. One of the reasons was that the severity of gastrointestinal dysfunction was less serious in the pilot study than in the latter. These results stimulated the interest to explore whether omega-3 fatty acids could reduce the mortality of sepsis. A recent systematic review including 17 RCTs found that, although omega-3 fatty acid supplementation could reduce both intensive care unit (ICU) length of stay and duration of mechanical ventilation, it had less effect on reducing the mortality of patients with sepsis or septic shock [25]. However, this review did not investigate the possible factors that could reduce mortality. Therefore, the present review combining data from previous RCTs was performed to address the issue.

## Method

### Inclusion criteria

#### Types of studies

RCTs were included irrespective of blinding, publication status, or language. Quasi-randomized trials and historically controlled clinical trials were excluded.

#### Types of participants

Male or female adult patients of any age or ethnic origin who were diagnosed with sepsis according to different criteria of different areas and age were included in the systematic review.

The diagnostic criteria required for inclusion in the review were according to different areas and ages.

Sepsis was defined according to the international guidelines [2]. Despite new diagnostic criteria of sepsis, most studies used criteria developed in 2012 [26]. Sepsis was defined as a proven or suspected infection that induced at least two of the four markers of systemic inflammatory response syndrome (SIRS), namely temperature more than 38°C or less than 36°C, heart rate more than 90 beats/min, white blood cell count more than 12 or less than 4 × 10^9^/L, or respiratory rate more than 20 times/min or PaCO_2_ less than 4.2 kPa. ARDS was defined according to the Berlin definition [27].

Any type of fish oil or other omega-3 fatty acid formula was included as a therapeutic in treating septic patients at any dose or administration regimen. Fish oil and other omega-3 fatty acids were compared with no intervention, placebo, and other conventional nutritional therapeutics.

#### Types of outcome measures

This review focused only on mortality.

### Search methods for identification of studies

The Cochrane Central Register of Controlled Trials (CENTRAL) on *The Cochrane Library* (Issue 1 2017), MEDLINE (1950 to March 2017), Embase (1980 to March 2017), and the Chinese BioMedical Literature Database (1978 to March 2017) were searched. The CKNI Chinese Paper Database (from 1994 to March 2017) and the Wangfang Chinese Paper Database (from 1994 to March 2017) were also searched. No language or date restrictions were applied. “Sepsis,” “acute respiratory distress syndrome,” “ARDS,” “systemic inflammatory reaction syndrome,” “SIRS,” and “multiple organ dysfunction syndromes,” were used as target disease searching terms. The words “n-3 fatty acid,” “polyunsaturated fatty acid,” “omega-3 fatty acids,” and “fish oil” were searched as interventions.

## Data collection and analysis

### Selection of trials for inclusion

Two reviewers (HC and WS) independently selected the trials by reading the titles and abstracts of the citations. Any potentially eligible studies were retrieved for further identification according to the prespecified selection criteria. Any disagreements were resolved by discussion with a third reviewer (ZY).

### Assessment of methodological quality

The guidance provided by the Cochrane Handbook [27] was followed for assessment. The items of assessment of methodological quality are listed in Table 1.

**Table 1:** Assessment of methodological quality

Allocation concealment	Low risk of bias	Randomization method would not allow investigator/participant to know or influence the intervention group before the eligible participant entered in the study.
	Unclear	Randomization stated but no information on the method used was available.
	High risk of bias	Methods of randomization used such as alternate medical record numbers or unsealed envelopes; any information in the study indicating that investigators or participants could influence the intervention group.
Blinding	Adequate	Blind to investigators, participants, outcome assessors, and data analysts.
	Inadequate	The treatment group could be identified in > 20% of participants due to side effects of treatment.
Incomplete outcome data	Low risk of bias	Specifically reported by authors that intention-to-treat analysis was undertaken; this was confirmed at the study assessment stage. If the analysis was not clearly stated but it was confirmed at the study assessment stage, it would also be granted a judgment of “Yes.”
	Inadequate	If the analysis was not clearly stated, or if it was stated but there was no confirmation that it had taken place at the study assessment stage, it would also be judged as “inadequate.”
	High risk of bias	No intention-to-treat analysis was reported with no confirmation at the study assessment stage.

### Data extraction

Data were extracted independently by two reviewers (HC and SW) using a self-developed data extraction form. Papers not in Chinese, English, Japanese, or German were translated with the help of the Cochrane Heart Group. The following characteristics and data were extracted from each included trial: primary author; study design and quality; mean age, gender, and ethnic origin of patients; number of randomized patients and number lost during follow-up; patient inclusion and exclusion criteria; dosage and duration of interventions, outcome measures; and number and type of adverse events.

Data on the number of patients with each outcome, by allocated treatment group, irrespective of compliance or follow-up, were sought to allow an intention-to-treat analysis.

### Data synthesis and statistical analysis

The dichotomous data were presented as relative risk (RR) and continuous outcomes as difference in means, both with 95% confidence intervals (CI). Intention-to-treat analysis was performed where possible. For dichotomous outcomes, patients with incomplete or missing data were included in a sensitivity analysis by counting them as treatment failures to explore the possible effect of loss to follow-up on the findings (a “worst-case” scenario). Also, a meta-analysis within subgroups was performed where individual trials included patients with similar baseline severity. Heterogeneity was analyzed using chi-squared test and *I*^2^ test [28]. *I*^2^ values of 25%, 50%, and 75% corresponded to low, medium, and high levels of heterogeneity, respectively. A fixed-effect meta-analysis was performed if no statistically significant heterogeneity existed among data from all trials. Otherwise, a random-effects meta-analysis was performed. RevMan 5.3 (Copenhagen: The Nordic Cochrane Centre, The Cochrane Collaboration, 2014) was used to carry out the meta-analysis.

Reduction of mortality (RM) between the treatment group and the control group was calculated and analyzed as an outcome. Other variables included total number of studies (TN), treatment duration, methods of nutrition support [enteral nutrition (EN) or parenteral nutrition (PN)], concentration ratio of *n*-6 to omega-3 fatty acids, and comparison methods. The latter four variables were analyzed in terms of their partial correlation coefficients and *P* values with RM when the other three factors were calculated as control variables. SPSS 17.0 for Windows (SPSS, IL, USA) was used for statistical analysis. If *P* value was less than 0.05, the correlations were supposed to be significant.

## Results

### Description of studies

**Fig. 1:**
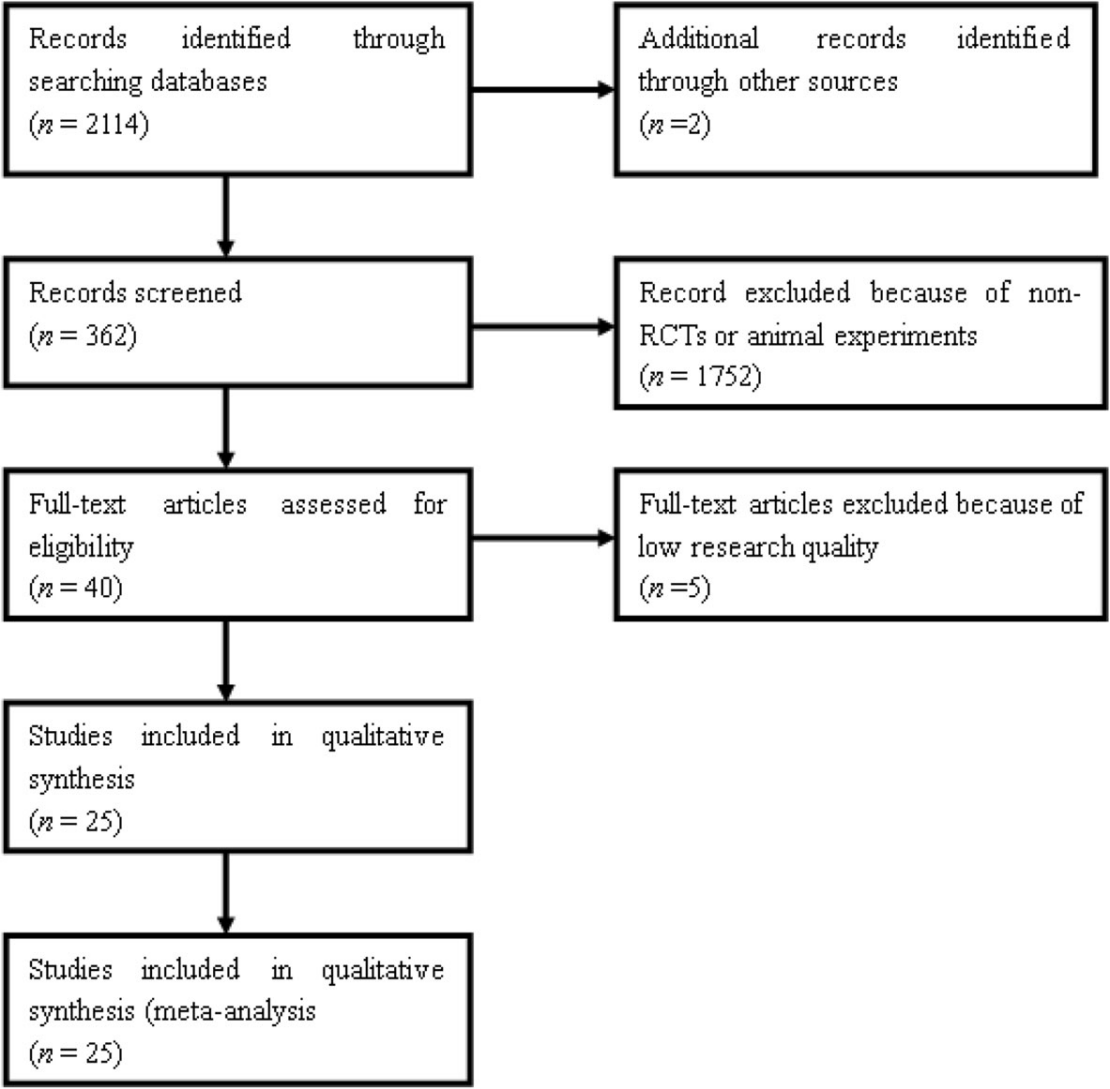
Flow diagram of included studies

The searches found 2114 records after duplicates were removed. Of these, 2066 papers were excluded because they were reviews, nonhuman research, controlled studies, or RCTs comparing different ablative methods. Of the remaining 48 full texts that were selected initially and read through, 40 RCTs were initially included (Table 1). However, only 25 of them with 2417 participants reported mortality in their studies (Fig. 1). The other six RCTs were excluded for reasons depicted in the characteristics of excluded studies. These studies were performed mainly in Asia (China, Japan), America (USA), Europe (German, Spain), and Africa (Republic of South Africa). Eighteen RCTs (in the 25 RCTs initially selected) reported higher APACHE II score or MODS assessment score (including SOFA and Marshall Scores) and were included to analyse independently.

### Risk of bias in included studies

**Fig. 2:**
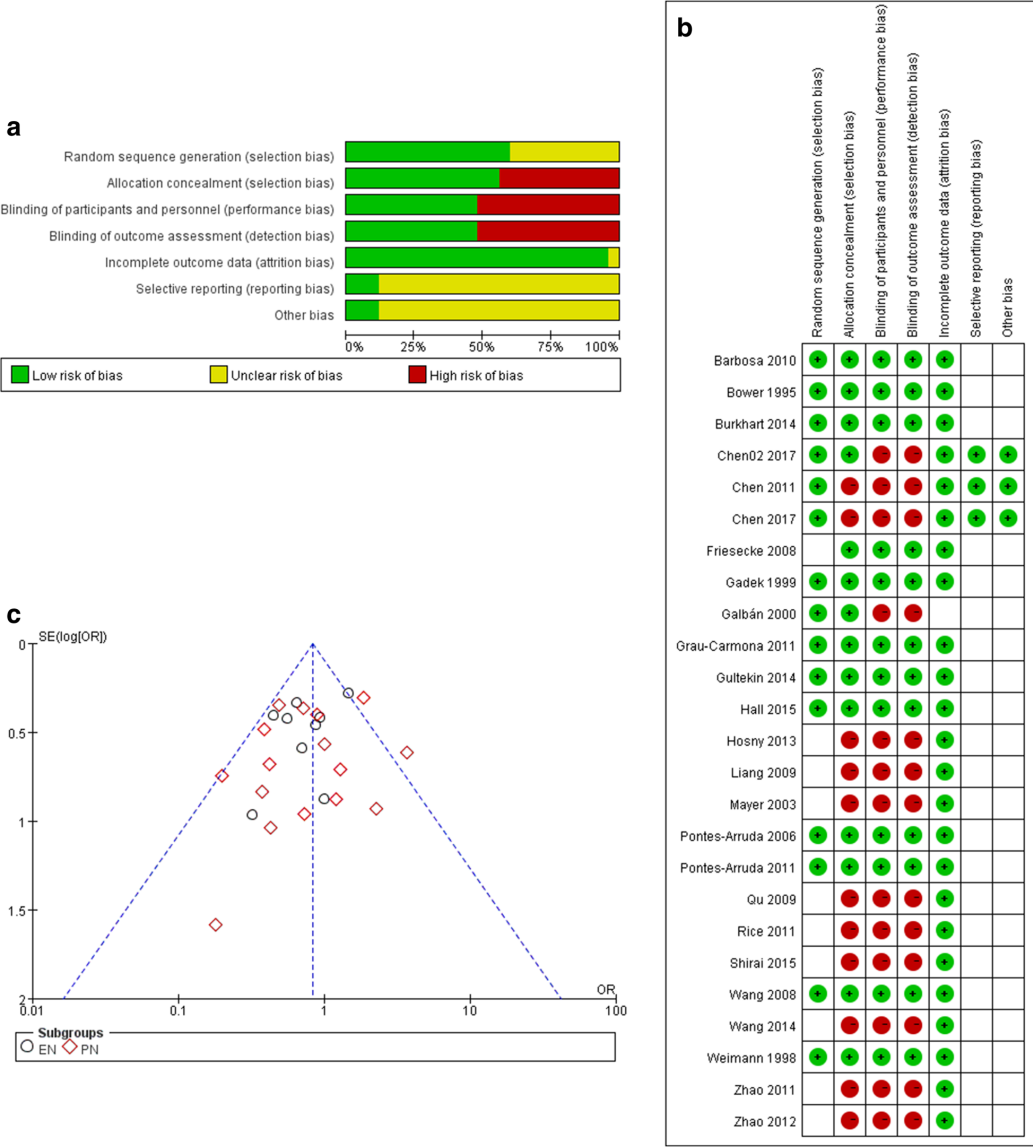
Risk of bias of included studies (**a**- total assessment of risk of bias. **b**- Risk list for each RCT. **c**- funnel plot of the included studies)

Selection bias: A total of 19 RCTs reported randomized methods, and 16 RCTs reported allocation concealment. Allocation concealment methods in 23 RCTs were supposed to be high risk. Among 25 RCTs reporting mortality, 15 RCTs depicted randomized methods and 14 RCTs reported allocation concealment with low risk (Fig. 2a and b).

Performance bias: 14 RCTs were double-blind studies. Blinding methods of other trials were with high risk. Twelve of 25 RCTs, which reported mortality, were low risk in their blinding methods.

Detection bias: Blinding to assessment was supposed to show low risk in 14 RCTs, while the remaining RCTs had high risk; 12 of these reported mortality.

Attrition bias: Thirty-eight trials reported incomplete outcome data or had no incomplete outcome. Among 25 RCTs reporting mortality, 24 RCTs had no attrition bias problems.

Reporting bias: Three RCTs conducted in the ICU of our institute reported all clinical and laboratory data so that they were supposed to be no selected report problem [21–23]. Other RCTs were unclear in selective reporting bias.

Publication bias: The studies are distributed closely with the 95% confidence interval axis (Fig. 2c).

### Baseline conditions of studies

Two RCTs included patients with severe acute pancreatitis, and one included severely injured patients in trauma, all of which caused sepsis-induced ARDS needing mechanical ventilators. Five RCTs included patients diagnosed with sepsis-induced ARDS. The remaining included sepsis patients. Among 25 RCTs that reported mortality rate, 16 listed the baseline Acute Physiology and Chronic Health Evaluation (APACHE) II score, and one reported the baseline APACHE III score. Eight RCTs reported the Sepsis-related organ failure assessment (SOFA) score, and three RCTs reported the baseline Marshall score (Table 2).

**Table 2:** Characteristics of included studies

Study/time	Cases(*n*/*N*)^a^	Critical score	Included patients	Treatment duration	Randomized methods	Blinding	Nutrition supplement in control group	Nutrition supplement in experimental group	Comparison method^b^
Wang 2014 [51]	53(25/23)	–	Severe abdominal infection-inducedd sepsis	5 days	Did not depict	No.	TPN application in 24–48 h after admission. Carole: 25 kcal/(kg ⋅ day), AA 1.2 g/(kg ⋅ day)	Plus Omegaven 0.2 g/(kg ⋅ day) based on nutrition in control group	A + B/A
Zhao 2012^c^ [52]	126(62/64)	APACHE II 21.1	Sepsis	7 days	Did not depict	No	Mixed feeding (specific unknown)	Omegaven 100 mL/day	A + B/A
Liang 2009^c^ [53]	148(70/78)	APACHE II 24.5	Pneumonia induced sepsis	7 days	Did not depict	No	Soybean fatty acid	Soybean fatty acid plus Omegaven 100 mL/day	A + B/A
Zhao 2011^c^ [29]	102(49/53)	APACHE II 21.1	Sepsis induced ARDS	7 days	Did not depict	No	Mixed feeding	Plus Omegaven 100 mL/day	A + B/A
Qu 2009^c^ [54]	40(20/20)	Mean APACHE II 17.7	Sepsis-induced gastrointestinal dysfunction	5 days	Did not depict	No	TPN 83.65 kJ/kg with nitrogen 0.2 g/kg	Plus Omegaven 1–2 mL/(kg ⋅ day)	A + B/A
Chen 2011^c^ [21]	30 (15/15)	Mean APACHE II 15.13/Mean Marshall Score 6.27	Sepsis-induced gastrointestinal dysfunction (with China diagnosis criteria)	7 days	Random-numbers table	No	LCT/MCT 20 kcal/(kg ⋅ day)	Plus Omegaven 100 mL/day	A + B/A
Chen 2017^c^ [22]	40 (20/20)	Mean APACHE II 23.4/Mean Marshall Score 9.8	Sepsis-induced gastrointestinal dysfunction (with China diagnosis criteria)	7 days	Random-numbers table	No	LCT/MCT 20 kcal/(kg ⋅ day)	Plus Omegaven 100 mL/day	A + B/A
Chen02 2017^c^ [23]	78 (41/37)	Mean APACHE II 32/Mean Marshall Score 9.3	Sepsis-induced gastrointestinal dysfunction (with Europe diagnosis criteria)	The whole duration in ICU.	Random-numbers table	No	LCT/MCT 20 kcal/(kg ⋅ day)	Plus Omegaven 100 mL/day	A + B/A
Burkhart 2014^c^ [55]	50 (25/25)	Mean APACHE II 26	Sepsis	7 days	Computer-based system	Yes (sealed envelopes)	Standard treatment (enteral/parenteral nutritional therapy but did not contain additional *n*-3 fatty acid)	Omegaven 0.2 g/(kg ⋅ day) or *n*-3 fatty acids 0.12 mg/(kg ⋅ day)	A/B
Hall 2015^c^ [56]	60 (30/30)	Mean APACHE II 19/Mean SOFA score 7.3	Sepsis (initiate within 12 h)	14 days or the whole duration if earlier	Random-numbers table	Yes (sealed envelopes)	Standard medical care only	0.2 g Omegaven/(kg ⋅ day) (0.05 g/(kg ⋅ h))	A/B
Rice 2011^c^ [30]	272 (143/129)	Mean APACHE III 93.8	Acute lung injury (ALI) Patients had to receive mechanical ventilation	21 days	2^a^2 factorial design	No	Control supplement	*n*-3 enriched enteral formula	A + B/A
Pontes-Arruda 2011^c^ [57]	115 (57/58)	Mean SOFA score 8.8	Early sepsis and required enteral nutrition	7 days	Web-based central randomization system	Yes (double blind)	Control enteral diet (ensure Plus HN), which did not contain DHA/EPA/GLA *n*-3:*n*-6 = 1:3.8	Study enteral diet (Oxepa) *n*-3:*n*-6 = 1:1.85	A + B/A + B
Grau-Carmona 2011^c^ [31]	132 (61/71)	Mean APACHE II 19/Mean SOFA score 9	Sepsis, mechanical ventilation; could receive EN	16 days	Computer-generated random-numbers table	Yes	Control diet *n*-6*:n*-3 = 5.8:1	EPA-GLA diet *n*-6*:n*-3 = 1.5:1	A + B/A + B
Barbosa 2010 [58]	25 (13/10)	–	SIRS or sepsis (who were predicted to need PN: severe pancreatitis, MODS, excisional surgery)	6 days	Random-numbers table	Yes (sealed envelopes)	50:50 mixture of an oil rich in medium-chain fatty acids Soybean (termed as MCT/LCT) Nutriflex Lipid Special	50:40:10 mixture of an oil rich in medium-chain fatty acids, soybean oil, and fish oil Lipolus, B. Braun, Portugal	A + B/A
Wang 2008^c^ [59]	40 (20/20)	Mean APACHE II 13/Mean SOFA score 7	Severe acute pancreatitis	5 days	Computer-derived block randomization	Yes (Double blind)	SO (Lipovenoes 20%; Fresenius, Germany)	FO-supplemented SO (Omegaven 10%; Fresenius, Germany) *n*-3:*n*-6 = 1:4	A + B/A
Friesecke 2008 [60]	166 (83/83) One in the control group was lost to follow-up.	–	Critical care patients with/without SIRS	7 days	Did not depict	Yes (double blind)	Lipofundin MCT (Deltamin, Deltaselect, Dreieich, Germany)	Omegaven (Fresenius Kabi) and Lipofundin MCT *n*-3:*n*-6 = 1:2	A + B/A
Pontes-Arruda 2006^c^ [32]	103 (55/48)	Mean APACHE II 19.5 /Mean SOFA score 5.5	Either severe sepsis or septic shock requiring mechanical ventilation	7 days	Did not depict	Yes (Double blind)	High-fat, low-carbohydrate enteral formulation *n*-3:*n*-6 = 3.8:1	Diet enriched with EPA, GLA, and enhanced levels of antioxidant vitamins. *n*-6:*n*-3 = 1.85:1	A + B/A + B
Mayer 2003^c^ [61]	21 (10/11)	Mean APACHE II 19.6	Sepsis and SIRS	5 days	Did not depict	No	Standard *n-*6 lipid infusion (Lipoven; Fresenius Kabi, Germany)	*n*-3 lipid infusion (Omegaven; Fresenius Kabi, Germany)	A/B
Galbán 2000 [62]	181 (94/87)	–	Sepsis, with APACHE II score more than 10	17 days	Computer-generated randomization program	No	High-protein control feed (Precitene Hiperproteico) 1414 ± 471 kcal/day	Enteral feed enriched with arginine, mRNA, and ω-3 fatty acid from fish oil 1231 ± 411 kcal/day	A + B/A + B
Gadek 1999 [33]	98 (51/47)	–	ARDS caused by sepsis/pneumonia, trauma, or aspiration injury	7 days	A permuted-block randomization design	Yes (double blind)	High-fat, low-carbohydrate enteral nutrition formula	EPA + GLA enteral diet	A + B/A + B
Weimann 1998 [63]	29 (16/13)	Mean APACHE II 6.5	SIRS and MODS after severe trauma	31 days	Did not depict	Yes (double blind)	Control	IMPACT formula with 10.5% omega-3 fatty acid (Sandoz Nutrition, Berne, Switzerland)	A + B/A
Bower 1995^c^ [64]	296 (167/159)	Mean APACHE II 19.2	Critical care patients, including severe trauma, sepsis, SIRS	7 days	Computer-generated randomization program	Yes (double blind)	Conventional nutrition formula (Osmolite HN, Ross Laboratories, OH, USA)	RNA, Mwer oil (Impact, Sandoz Nutrition, MN, USA)	A + B/A + B
Gultekin 2014 [65]	32 (16/16) With mean age of 62.9 ± 12.2 years	–	Sepsis	5 days	Did not depict	Yes (double blind)	Olive oil containing standard lipid emulsion (1.3 ± 0.1 g/(kg ⋅ day))	TPN: 80% olive oil + 20% soy oil + 10 g fish oil (10% Omegaven, Fresenius Kabi, Germany)	A + B/A
Shirai 2015^c^ [34]	46 (23/23)	Mean APACHE II 24/Mean SOFA score 10	Sepsis-induced ARDS	14 days	Did not depict	Yes (single blind)	A standard isocaloric enteral diet	Enteral diet enriched with EPA, GLA, and antioxidants	A + B/A + B
Hosny 2013^c^ [66]	75 (25/25/25) High-dose group = 25 Control group = 25	Mean SOFA score 3.7	Early stage of sepsis	7 days	Did not depict	No	Conventional sepsis treatment	High-dose omega-3 fatty acids; antioxidants in the form of ascorbic acid 1000 mg/day	A/B

^a^Cases: the total samples; *n*: samples in the treatment group; *N*: samples in the control group

^b^Comparison methods: A/B: directly compared two types of nutrition with or without *n*-3 fatty acids. A + B/A: comparison of *n*-6 fatty acids plus *n*-3 fatty acids to *n*-6 fatty acids alone. A + B/A + B: comparison of two types of nutrition with various concentrations of *n*-6 and *n*-3 fatty acids

^c^RCTs including patients with APACHE II score more than 15, or SOFA score more than 2 were considered as similar baselines

In 25 RCTs included in mortality analysis, nine compared variable EN with different concentrations of omega-3 fatty acids, while the other 16 compared different types of PN. In the PN subgroup, three studies compared soybean oil to omega-3 fatty acids directly (A vs B), and other 13 studies compared soybean oil plus omega-3 fatty acids to soybean oil alone (A + B vs B). All characteristics of studies are shown in Table 2.

### Effects of the interventions

#### Total mortality

**Fig. 3:**
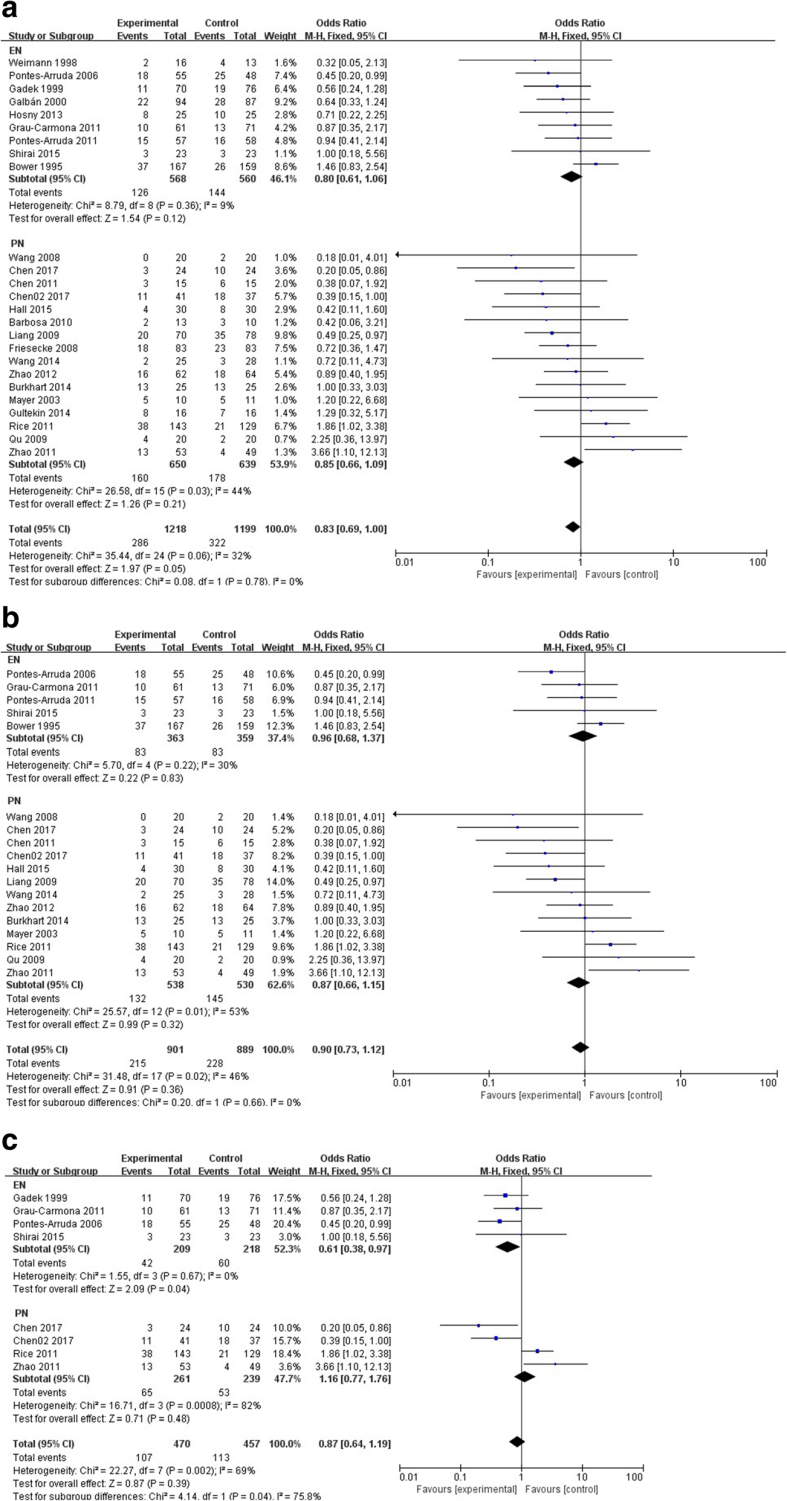
Comparison of mortality between omega-3 fatty acid group and the control group (**a**- Comparison of total mortality. **b**- Comparison of mortality in studies with similar baseline. **c**- Comparison of mortality in studies with sepsis-induced ARDS)

A total of 25 RCTs were included in mortality analysis (Fig. 3a).

Nine RCTs with 1128 patients compared different types of EN with various concentrations of omega-3 fatty acids for treating sepsis and ARDS. Further, 126 patients in the omega-3 fatty acid group (22.18%) and 144 patients in the control group (25.71%) died at the end of the observation period. Another 16 RCTs including 1289 patients compared PN with the conventional treatment of sepsis. Moreover, 160 patients in the omega-3 fatty acid group (accounted for 24.62%) and 178 patients in the control group (27.86%) died at the end of observation period. The heterogeneity among studies in each subgroup (EN and PN) was low. The *I*^2^ value was 0% in the EN group and 40% in the PN group. A fixed-effects model was used for meta-analysis. The *P* value was 0.12 and 0.20, respectively, which demonstrated that omega-3 fatty acids had no significant effect on reducing the mortality of sepsis and ARDS.

#### Mortality of studies with similar baseline

Data from 18 RCTs including 1790 patients with severe sepsis and sepsis-induced ARDS having similar severities were analyzed (Table 2, Fig. 3b).

Five RCTs with 722 patients were included in the EN subgroup. Further, 83 patients died in the high- and low-concentration omega-3 fatty acid subgroups, respectively. The heterogeneity among studies was low (*I*^2^ = 27%), and mortality was insignificantly low in the high-concentration omega-3 fatty acid subgroup (22.87% vs 23.12%, *P* = 0.24).

Thirteen RCTs with 1068 patients were included in PN subgroup. The heterogeneity among studies was medium (50%). A fixed-effects model was used for analysis. Moreover, 132 patients in the omega-3 fatty acid group (24.54%) and 145 patients in the control group (27.36%) died at the end of the observation period (*P* = 0.32).

Eight RCTs including 927 patients with sepsis-induced ARDS. Among these RCTs, six trials clearly indicated that they included patients with ARDS [29–34]. In another two trials, although we included patients with sepsis-induced gastrointestinal dysfunction, all patients were involved in severe respiratory failure and ARDS which needed mechanical ventilators [22, 23]. Then these two RCTs were also included. Other studies were excluded because it was not possible to determine whether they included ARDS patients or not. Omega-3 fatty acids did not show positive effect (*p* = 0.39). But in EN subgroup, omega-3 fatty acids treatment seemed to have some benefits in reducing mortality rate (*p* = 0.04). The results were seen in Fig. 3c.

#### Partial correlation analysis

**Fig. 4:**
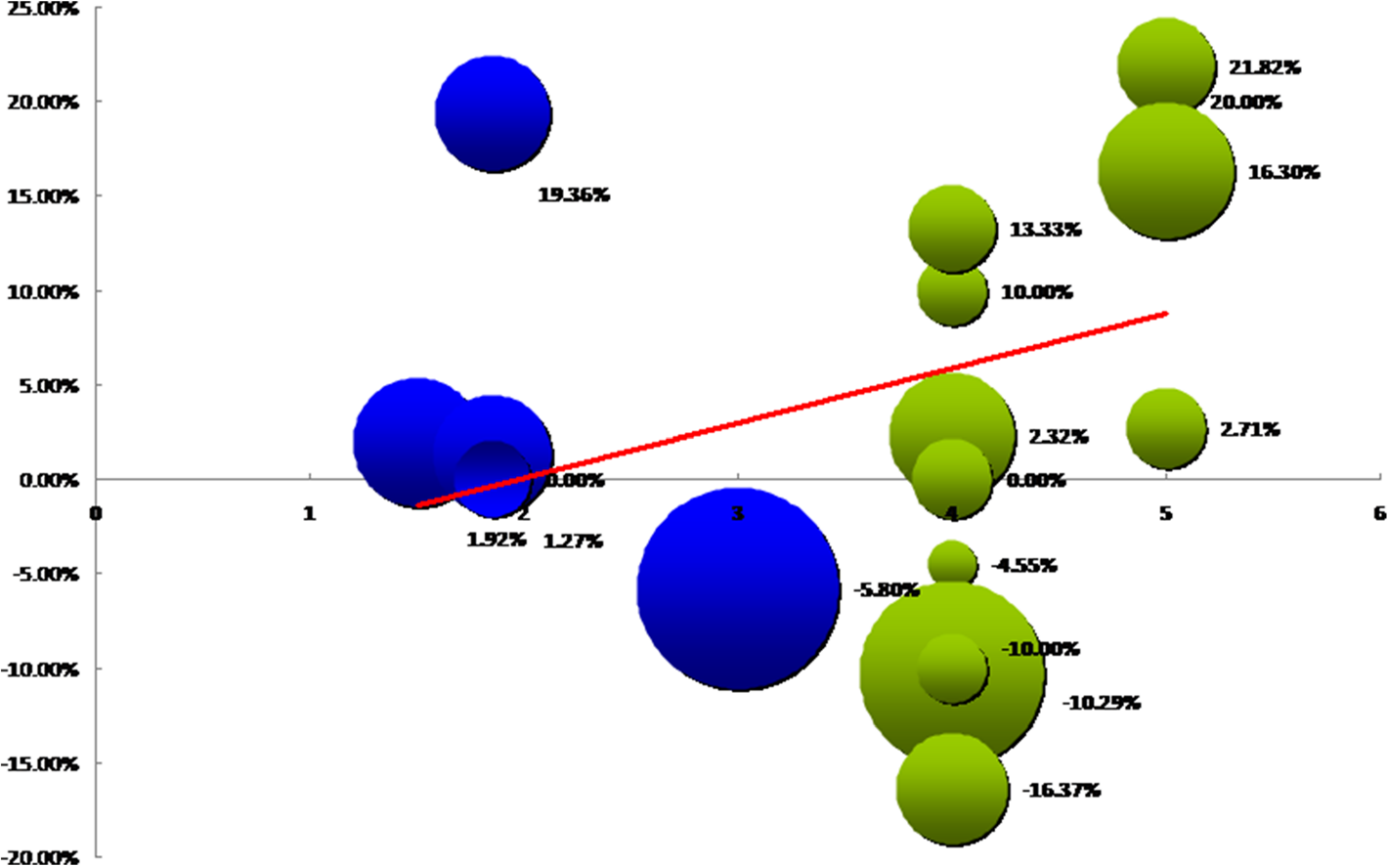
Partial correlation analysis of studies’ characteristics and reduction of mortality (RM)

The results demonstrated that in 25 RCTs observing mortality, five factors, namely concentration ratio of *n*-6 to omega-3 fatty acids, treatment duration, methods of nutrition support, comparison methods, and TN, had no correlation with RM. However, when data from RCTs including patients with similar severity were analyzed, the concentration ratio of *n*-6 to omega-3 fatty acids was found to have a positive correlation with RM (*γ* = 0.60, *P* = 0.02), whereas the total number of each RCT had a negative correlation with RM (*γ* = − 0.54, *P* = 0.05). Other factors had no correlation with RM (Table 3 and Fig. 4).

**Table 3:** Partial correlation analysis of mortality and its influencing factors

	All RCTs that emphasized mortality		RCTs that included patients with similar baseline	
	Reduction of mortality		Reduction of mortality	
*n*-6:*n*-3	*γ*^a^ = 0.23	*P* = 0.31	*γ* = 0.60	*P* = 0.02
Treatment duration	*γ* = 0.12	*P* = 0.59	*γ* = 0.24	*P* = 0.41
Nutrition support methods	*γ* = − 0.26	*P* = 0.26	*γ* = − 0.01	*P* = 0.99
Comparison methods	*γ* = − 0.19	*P* = 0.40	*γ* = − 0.48	*P* = 0.08
TN^b^	*γ* = − 0.32	*P* = 0.16	*γ* = − 0.54	*P* = 0.05

^a^*γ*: partial correlation coefficient. ^b^*TN* total number of patients in the study

## Discussion

This systematic review was conducted to evaluate the effect of omega-3 fatty acids on the mortality of sepsis and ARDS. Forty RCTs were initially included, but only 25 of them assessed mortality. A total of 2417 patients were involved in these RCTs, and 608 patients died (accounted for 25%) at the end of study duration. Omega-3 fatty acids seemed to have no effect on reducing mortality when data from all these studies were combined. It was believed that severities affected the final result. However, the difference in mortalities between the treatment and control groups was also insignificant when data only from RCTs including severe sepsis and ARDS with similar severity were analysed. Although EN seems to reduce the mortality of sepsis-induced ARDS patients, the results only come from four small studies. Generally speaking, omega-3 fatty acids have no significant effect on sepsis-induced ARDS. Furthermore, concentration ratio of *n*-6 to omega-3 fatty acids, treatment duration, nutrition support methods, comparison methods, and total number of studies were set as coefficient variables, and their correlation with RM in the RCTs was calculated. The results found that in the RCTs including patients with similar severity, the concentration ratio *n*-6 to omega-3 fatty acids seemed to have a positive correlation with RM, and the total number of studies had a negative correlation with RM.

Animal experiments demonstrated the anti-inflammatory effect of omega-3 fatty acids in sepsis [35]. A cecal ligation and puncture model of sepsis was established, and fish oil supplementation and soybean-based total parenteral nutrition (TPN) were administered. Acute lung injury score, immunity, and inflammation in rats were observed in fish oil–supplemented TPN group [36]. Long treatment duration (28 days) of omega-3 fatty acids could improve the survival of mice with *Staphylococcus*-induced sepsis [37]. Some researchers have raised objections to this conclusion. The results from a recent study on rodents suggested that although *n*-3 polyunsaturated fatty acids had an antioxidant effect, they might worsen septic shock–induced vascular dysfunction, and not improve the survival rate of the disease [38]. The results from clinical trials had similar conclusions to these animal experiments. In the three RCTs conducted by the ICU, one of them had negative results in improving survivals while the other two had positive results in reducing mortality at the end of observation period [21–23]. However, even the latest RCT that observed that 60-day mortality could be reduced in the fish oil group compared with the control group could not eliminate the suspicion [23]. In 2016, a meta-analysis combined data from 11 RCTs and concluded that omega-3 fatty acids had no effect on reducing the mortality of sepsis [39]. Although the meta-analysis searched the PubMed database alone, its result was similar to that of the present review. Several pieces of evidences show that omega-3 fatty acids have anti-inflammatory effects [13]. Inflammation is a critical link of sepsis [14]. Therefore, it can be concluded that the anti-inflammatory characteristics of omega-3 fatty acids are not strong enough to reduce the mortality of sepsis. In fact, sepsis is a more complicated syndrome. Its mortality might be affected by many other factors such as tissue perfusion, antibiotics, organs support measurements, and so on. Omega-3 fatty acids could only provide partial effects in improving the conditions of the disease, rather than directly reducing the mortality.

Some factors such as nutrition supplementation, concentration ratio n-6 to n-3 fatty acids, and interventional comparison may affect the impact of omega-3 fatty acids in treating sepsis or ARDS. Omega-3 fatty acids can be applied through enteral and parenteral routes. Recently, PN has been shown to be insufficient in improving the prognosis of critically ill patients [40]. A study including infant patients observed that the application of soybean oil PN would induce a high risk of essential fatty acid deficiency [41]. However, fish oil may change this condition. Animal experiments discovered that parenteral rather than enteral fish oil supplementation had additional effects on reducing Bax mRNA expression, increasing Bcl2 mRNA expression, promoting proliferation of intestinal epidermal cells, lengthening the villus, and inhibiting apoptosis of cells [42]. On the contrary, a study found that Leukotriene B4 (LTB4) and Thromboxane B2 (TXB2) reduced the increase to the pretreatment level when the enteral supplement was suspended [43]. No evidence exists demonstrating that enteral supplements of Fish oil could improve the prognosis of severe inflammatory reaction [44]. These evidences might imply that parenteral supplementation of omega-3 fatty acids would be better than enteral supplementation. However, a significant difference between parenteral and enteral supplementation in improving survival rates was not observed in the present review.

A certain proportion of n-3 and n-6 fatty acids is specified to be used for anti-inflammatory effect. Previous researches suggested that 1:2 –1:4 would be a better proportion for eliminating the inflammatory reaction [45]. The present review found a positive correlation between concentration and reduction of the mortality of severe sepsis and ARDS. It means that the higher the ratio of *n*-6 to omega-3 fatty acids, the more the RM. On the one hand, this result implied that the ratio was not the higher the better. On the other hand, it has been found that the concentration ratio of *n*-6 to omega-3 fatty acids in the RCTs is equipped with a certain standard. It should be noted that omega-3 fatty acids alone may not play an anti-inflammatory role. Therefore, a comparison of *n*-6 and omega-3 fatty acids alone may not make sense.

Research methodologies of most studies are suitable. However, still some RCTs have a high risk of bias. The most common bias is allocation concealment. Half of these studies were single-blind trials. However, how this bias affects the mortality is still unknown. One of the interesting results is that the total number of studies has a negative correlation with RM. It means that the larger the scale of the study, the less the RM will be. The estimation of sample size in clinical trials is one of the most important issues in the research design. In general, the larger the research scale, the closer the result. On the contrary, an increase in the number of subjects may lead to more wastage of resources [46]. Almost all studies did not estimate the included sample. In one of the studies [23], the sample was estimated in accordance to a previous pilot study [22]. The significant correlation between study sample size and RM may be only statistical. However, it is also possible that the increase in sample size does reflect the fact that omega-3 fatty acids have no significant effects on reduction in mortality.

Although omega 3 fatty acid is a kind of fat preparation, no evidence demonstrates that it could increase blood lipid. On the contrary, studies suggest that fish oil can reduce serum triglyceride (TG) [47], and the low-density lipoprotein (LDL) as well [48], which seems to be little influence by cholesterol and high-density lipoprotein (HDL). In our previous study, we also observed total bilirubin and fibrinogen before and after treatment of omega 3 fatty acid [49]. After seventh days of treatment, the total bilirubin in the fish oil treatment group was lower than that of the control group, while fibrinogen of two groups was similar. At present, there is lacking of evidence that omega 3 fatty acid can deteriorate liver function. For children with intestinal dysfunction associated with liver dysfunction, treatment with fish oil can reduce inflammatory related indicators and bring potential benefits. However, whether fish oil can be applied to patients with liver dysfunction and whether the dose is in dispute or not are still on controversial [50].

## Conclusions

Omega-3 fatty acid supplementation could reduce the mortality rate of sepsis and sepsis-induced ARDS, but this finding is still not supported by most evidence. Hence, investigating based on suitable concentrations and indications may be the directions of future research.
